# Spatio-temporal skin strain distributions evoke low variability spike responses in cuneate neurons

**DOI:** 10.1098/rsif.2013.1015

**Published:** 2014-04-06

**Authors:** Vincent Hayward, Alexander V. Terekhov, Sheng-Chao Wong, Pontus Geborek, Fredrik Bengtsson, Henrik Jörntell

**Affiliations:** 1 Sorbonne Universités, UPMC Univ Paris 06, UMR 7222, ISIR, F-75005, Paris, France; 2 Neural basis for Sensorimotor Control, BMC F10, Lund University, SE-22184 Lund, Sweden

**Keywords:** contact mechanics, spatio-temporal skin stimulation, cuneate nucleus

## Abstract

A common method to explore the somatosensory function of the brain is to relate skin stimuli to neurophysiological recordings. However, interaction with the skin involves complex mechanical effects. Variability in mechanically induced spike responses is likely to be due in part to mechanical variability of the transformation of stimuli into spiking patterns in the primary sensors located in the skin. This source of variability greatly hampers detailed investigations of the response of the brain to different types of mechanical stimuli. A novel stimulation technique designed to minimize the uncertainty in the strain distributions induced in the skin was applied to evoke responses in single neurons in the cat. We show that exposure to specific spatio-temporal stimuli induced highly reproducible spike responses in the cells of the cuneate nucleus, which represents the first stage of integration of peripheral inputs to the brain. Using precisely controlled spatio-temporal stimuli, we also show that cuneate neurons, as a whole, were selectively sensitive to the spatial and to the temporal aspects of the stimuli. We conclude that the present skin stimulation technique based on localized differential tractions greatly reduces response variability that is exogenous to the information processing of the brain and hence paves the way for substantially more detailed investigations of the brain's somatosensory system.

## 1. Introduction

The mammalian skin is endowed with a rich set of receptors that respond to mechanical stimuli induced by its interaction with objects [1,2]. A tactile event, even brief, can result in the activation of thousands of receptors. The time-course of activation of these receptors, their mechanical tuning and their locations in the skin provide information that is integrated by the brain to build a percept of the event, but little is known about the underlying mechanisms.

We wondered whether it was possible to reliably evoke reproducible spike responses from complex spatio-temporal stimuli in the skin. To test this hypothesis, we recorded the spike activity of single neurons in the cuneate nucleus of the cat in response to parametrized families of spatio-temporal stimuli created by complex distributed tractions gradients at the surface of the skin. Reproducibility of the stimulus generation was defined by the property that for all tested neurons, a given stimulus elicited responses drawn from the same underlying distribution.

Most skin receptors are mechanoreceptors that respond to strain induced in the tissues; therefore, a stimulation technique must aim to impose well-controlled skin strain distributions. Mechanized approaches to the stimulation of the somatosensory system have, by and large, relied on moving probes and counter-surfaces against a digital pad using servo-controlled mechanisms that track preset position or force trajectories. These counter-surfaces can be flat, have features such as gratings, edges, or have raised elements of various geometries, and the movements can be normal or tangential to the pad surface [3–10]. Even if the movements of the counter-surface are commanded precisely and the digit is immobilized, the evolution of skin deformation cannot be precisely controlled, owing to complex visco-elastic tissue mechanics [11–13], and to the biotribology responsible for the interfacial forces during adhesion and sliding [14–16]. One approach to counteracting the lack of stimulation reliability is to monitor the region of interaction through a transparent surface in order to relate local skin deformation to neuronal responses [4].

**Figure 1.**
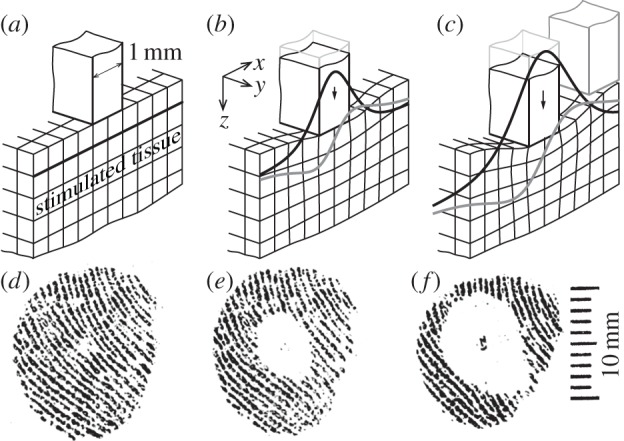
Non-local effects of indentation on subsurface strain. (*a*) Undeformed half-space with zero initial subsurface deformation (thick black line). (*b*) Upon downward normal indentation by a punch, the region surrounding the contact deforms as described by the classic Boussinesq–Flamant's problem that treats the deformation of a solid half space under the action of a line load. At a small distance beneath the surface, assuming Hooke's law [22], that is a linear approximation, the compression strain distribution follows the pattern shown by the black line and the shear strain distribution the pattern shown by the grey line. Actual punches produce finite contact surfaces and tissues exhibit non-ideal contact mechanics, yet the character of the functions that describe subsurface deformation is unaffected by variations in the geometry of the punch [23], and by the inhomogeneity and anisotropy of actual tissues [24]. (*c*) It is estimated that deformation extends to a region as large as 6 mm in diameter for a 1 mm of indentation in human fingers [25]. (*d*) Human fingerprint imaged with the technique described in [26]. (*e*) Same fingerprint with a 1 mm cylindrical punch indenting the skin by 1 mm. The deformation extends to a 6 mm region as evidenced by the loss of contact with the imaging surface, corroborating the results of [25]. (*f*) The region of deformation with an identation of 2 mm grows to almost 10 mm.

A significant advance toward the automated generation of stimuli was the development of an indenting probe actuated by feedback-controlled, moving-coil motors or by piezoelectric transducers [17]. With this technique, the responses of single afferents could be compared systematically in terms of skin punctual indentation amplitude and rate [18]. Von Frey hair stimulators have been automatized but their mechanical principle restricts their application to quasi-static stimuli [19]. An important evolution of this technique was a dense array of indentors interacting with the skin that combined stimulus variations in time with variations in space [20,21]. Nevertheless, indentation is associated with non-local contact mechanics that couple the effect of the movement of each probe with that of its neighbours, as shown in figure 1*a*–*c*, and which also depend on the normal load. In a fingertip, the lack of tissue homogeneity combined with the absence of reliable support (Dirichlet boundary condition, where the displacement of the boundary of a domain is imposed) in the vicinity of indentation, can also induce responses in mechanoreceptors located at considerable distances from the locus of stimulation [27]. The illustration of one the effects of non-local mechanics in the skin is shown in figure 1*d*–*f*.

As an alternative to punctual indentation, the skin stimulation technique that we used relied on localized strains induced by a large region of support divided into multiple small surfaces moving individually sideways. We synthesized a family of spatio-temporal stimuli and evaluated the activation of the mechanoreceptors in the skin through recordings from neurons of the cuneate nucleus, where first-order integration of tactile information takes place. We demonstrated that specific spatio-temporal stimuli elicited very low variability spiking patterns, indicating reliable activation of populations of skin sensory units from trial to trial. Our findings suggest that this technique can overcome the uncertainties resulting from complex skin tissue mechanics, enabling systematic studies of the neural correlates of tactile sensory inputs in the form of complex spatio-temporal stimuli.

## 2. Experimental methods

### 2.1. Stimulation by differential traction

**Figure 2.**
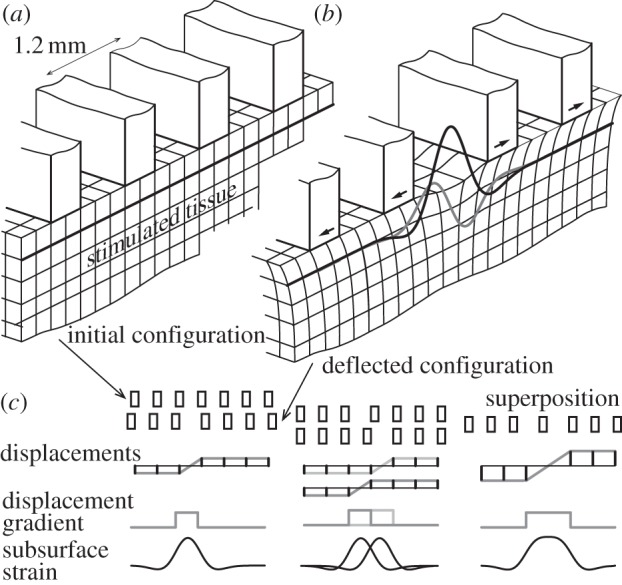
Synthesis of arbitrary strain distributions. (*a*) Undeformed half-space where the stimulated tissue is supported by a multiplicity of contact surfaces. (*b*) Relative lateral displacement of the adjacent surfaces induces a deformation pattern confined to the gap region similar in magnitude (black and grey lines) to single punch indentation, while neighbouring contact surfaces provide a uniform surface of support. (*c*) Synthesis by superposition of elemental stimuli. In the first column, the top row shows the initial configuration of a set of traction surfaces, the second row shows how they can be deflected, the third the corresponding displacements in ordinate, the fourth the surface displacement gradient and the fifth the resulting elongation strains beneath the surface. The second column is an exemplification of stimulus synthesis where a second pattern, similar to the first but shifted right one place, is superposed to the first. The third column shows how, with proper spacing [30], the stimulus resulting from the superposition of two elemental stimuli achieves a wider region of uniform strain, under the assumption of homogeneous material properties.

An array of contact surfaces produced differential traction distributions on the skin surface [28]. This approach exploits the similarities that exist between the strain fields produced by indenting line loads with those produced by differential lateral tractions at a small distance below the surface of a solid half space (see appendix A and [29]). The crucial point is that subsurface strains are confined to the regions between the contact surfaces as a result of their differential action, as indicated by figure 2*a*,*b*. This stimulation method takes advantage of the fact that strain is related to the gradient of displacement [22], that is, of the relative displacement of contiguous portions of an identical body. Crucially, the technique affords a uniform surface of support for the stimulated object that results in subsurface strains that are, by-and-large, independent from the net normal load and from the activation of neighouring traction surfaces. For small strains, linear superposition applies, which makes it possible to specify, within the limit of the assumptions of linearity and perfect contact, arbitrary spatio-temporal strain distributions as shown in figure 2*c*.

### 2.2. Test stimuli

**Figure 3.**
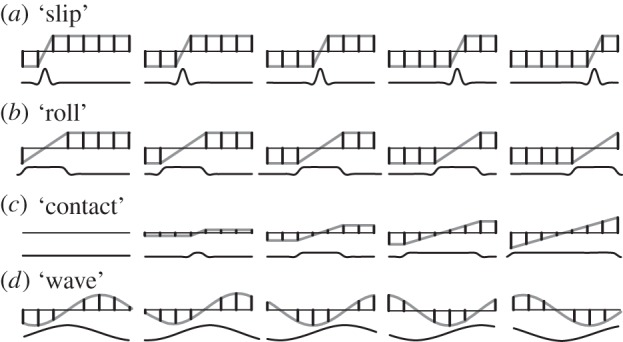
Stimuli afforded by combinations of elemental stimuli obtained by deflection of traction surfaces. (*a*) Region of concentrated strain moving from left to right, reproducing the effects of an edge slipping across the stimulated pad. (*b*) Similar stimulus but with a wider region of uniform strain that would be typical of a rolling contact. (*c*) Stimulus that is representative of a surface compressing a pad where the contact region grows in size, progressively recruiting strained tissue. (*d*) Progressive wave pattern that would result from sliding against an undulating surface.

A collection of activation patterns were designed to compose a typology of spatio-temporal stimuli differentiated by their size and by their mode of evolution. The simplest stimuli were created by maximally straining the smallest possible region. Similar strain distributions are induced by local features present on counter-surfaces having a small radius of curvature, similar to a sharp raised edge [31]. Slip on an otherwise smooth surface induces the motion of such features on the skin (figure 3*a*). By contrast, a counter-surface of radius of curvature greater than the contacting pad, for instance a flat surface, gives rise to a wider region of strain that also can move under a rolling contact condition (figure 3*b*). The formation of a contact (respectively, its termination) is characterized by progressive recruitment (respectively, release) of strained tissue (figure 3*c*). The stimulus corresponding to a moving grated surface results from sampling a travelling wave pattern, as in figure 3*d* [30]. For later reference, these four stimuli were labelled ‘slip’, ‘roll’, ‘contact’ and ‘wave’, respectively, and were all parametrized by a speed.

Comparable stimuli were all employed in past micro-neurographic studies [4,10,32,33], because they reflect simplified mechanical conditions that are applicable when there is large displacement of a probe relatively to an object [34]. We also used a stimulus, called ‘quadrant’, that activated fixed regions of the stimulator array. Subsets of three-by-three contiguous regions of skin were stimulated by repeated ‘stretch–hold–relax’ sequences applied at a rate of 4 s^−1^. The strains produced in the corresponding regions of the skin strongly activated the local receptors, which was useful to map the receptive field of a recorded neuron and verify that the field was centred within the active surface of tactile stimulator array. Finally, a control stimulus, termed ‘randomized’, was created by activating the traction surfaces with the same amplitudes as with the ‘wave’ stimulus but with a random permutation of the spatial order. This stimulus strongly activated the skin receptors but with no specific spatio-temporal organization.

### 2.3. *In vivo* stimulation

**Figure 4.**
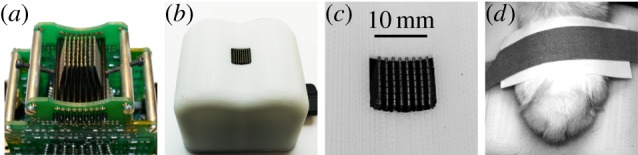
Stimulator device (Tactile Labs Inc.). (*a*) Stimulator head in (*b*) protective cover. (*c*) View of active surface and (*d*) stimulated paw. (Online version in colour.)

We employed a miniaturized stimulator device, shown in figure 4*a*, which is further described in appendix B and in [30]. The lateral movement of the 64 contact surfaces was produced through the bending action of piezoelectric actuators which for the purpose of this experiment were activated column by column. The device was encased in a protective cover (figure 4*b*) with a 1 cm^2^ window opening for the active surface (figure 4*c*). The targeted digital pads were coupled to the active surface by simple contact and by securing the paw with adhesive tape as shown in figure 4*d*.

The display head was driven through a time-division protocol implemented in a field programmable gate array circuit able to refresh the display each 1.5 ms. To ensure accurate signal reconstruction and owing to the wide-bandwidth response of the piezoelectric actuators, each channel signal processing chain was terminated by a low-pass analogue filter with a 100 Hz cut-off frequency. The configuration also included a dedicated Ethernet interface running under the User Datagram Protocol that enabled the device to be driven by a laptop computer. The entire system latency, 0.58± ms (*n* = 10, ±s.d.), was determined by measurement of the delay between the transmission of a packet (signalled by a strobe signal) and the mechanical movements of the contact surfaces (measured directly). This delay value was used to correct the alignment of stimuli and the neural responses in time.

### 2.4. Neuronal recordings

We recorded the spiking responses of neurons of the cuneate nucleus in the cat (appendix C) and focused on cells having their receptive fields located on the glabrous skin of the pads of the second and third digits, or on the central pad. The cuneate nucleus is a structure in the lower brainstem in which the neurons receive monosynaptic inputs from the primary afferents that innerve the skin mechanoreceptors [35]. It is organized somatotopically where inputs from the digits are represented in a separate population of neurons, see [35] for references. The results of a recent study indicate that the output of a cuneate neuron corresponds to an integrated signal from a limited number, of the order of 10 primary afferents [36]. As the preparation was a decerebrated animal, cortical processing had no influence on the activity of the cuneate neurons. Mechanical events in the skin activate the mechanoreceptors, and the primary afferent axons conduct the signals from the periphery to the central nervous system via the cuneate nucleus.

Once the location of the cuneate cell receptive field had been centred on the display, a period of spontaneous firing activity was recorded before starting the trials. The spontaneous activity between trials was continuously recorded to verify that the inter-trial period was sufficiently long to allow the spontaneous activity to return to its original value after at least 600 ms. Because the primary afferents make monosynaptic contact with cuneate neurons, the return to the background synaptic activity was an indication that the primary afferents that innervated the stimulated skin area also returned to resting levels and that the neurophysiological effects did not transfer from one trial to the next. It also argues against the possibility of the effect of mechanical hysteretis in the skin lasting longer than the inter-trial intervals.

For each cell, complete experimental sessions comprised combinations of stimuli patterns that were each repeated 50 times. The ‘slip’ stimuli, as in figure 3*a*, were synthesized so they moved at 10, 20, 40, 80 and 160 mm s^−1^. The ‘roll’ stimuli, as in figure 3*b*, moved at 5, 10 and 20 mm s^−1^. The ‘contact’ stimuli were, as in figure 3*c*, synthesized to simulate a ‘contact on’, with the borders diverging at 10, 20, 50, 100 and 200 mm s^−1^ from the centre. The ‘contact off’ stimuli were synthesized by reversing this pattern in time with the borders converging from the edges toward the centre of the active surface. A summary of the stimuli is presented in table 1. Sessions also comprised presentations of ‘wave’ stimuli that are not analysed in this report and a few repetitions of ‘randomized’ stimuli to provide a baseline response. Sessions were initiated once the mechanical stability of the pad-stimulator contact and the proper location of the receptive field of the cell within the active surface be verified with the ‘quadrant’ stimulus. We recorded *n* = 18 cells that lasted long enough to respond to 50 repetitions of 18 distinct stimuli, totalling 16 200 trials.

**Table 1.** For each stimulus, fractions in 1/1000th where the two-sample KS test reached an uncorrected threshold of *p* = 0.05, which was used as a criterion to decide that the two pooled neural responses were drawn from different distributions. Last line lists the mean numbers of spikes evoked by each stimulus across all cells.

stimuli	‘slip’					‘roll’			‘contact on’					‘contact off’				
velocity	10	20	40	80	160	5	10	20	10	20	50	100	200	10	20	50	100	200
KS median	6.0	0	0	0	0	9.0	2.0	6.0	4.0	0	0	0	1.0	3.0	0	0	0	0
KS upper bound^a^	12.2	0.8	0.1	0.1	0	8.3	3.0	4.1	2.1	0.4	0.2	2.5	0.6	4.5	7.6	6.7	7.5	7.1
KS highest value	18.4	1.9	7.1	1.8	2.8	37.9	32.9	17.9	7.7	17.7	22.5	17.6	3.9	13.1	24.3	24.7	28.0	47.0
mean spike count	114	83	61	38	21	305	168	92	56	33	20	14	9	61	35	22	14	7

^a^Of the 95% CI given by the Wilcoxon signed-rank test.

### 2.5. Similarity analysis

To substantiate the observation that the stimuli elicited consistent responses, we employed the two-sample Kolmogorov–Smirnov (KS) test to assess the response similarities for all stimuli and for all cells. This test takes a pair of data samples as input and tests the null hypothesis that they are drawn from an indentical, yet unknown, distribution. The samples comprised the time sequences, 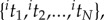 of the spike times measured from the beginning of the trial, where 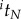 is the time of the last spike, *N*, of trial *i*. Pairs of samples were produced by pooling sequences from two randomly selected but disjointed subsets of trials of cardinality 24 for the same neuron and the same stimulation condition, omitting the first trial. If a neuron had a reproducible response to a stimulus, then there should be no statistically significant difference between any two samples constructed this way. Samples were deemed to be different if the probability computed by the KS test was below a significance level of 0.05. The procedure was repeated 1000 times for every neuron and every condition, and the fraction of cases when the two samples were significantly different were reported.

The similarities test reflected the similarities of the samples of spiking times drawn from unkown probability distributions. However, if the neurons responded randomly, ignoring the stimuli, then the spiking probability distributions could also be similar. To control for this unlikely possibility, all the neuronal responses were compared to a uniform distribution using the one-sample KS test. Moreover, if the spike responses were random yet non-stationary, the main test would show significant differences. Individual pooled data samples were produced by combining the spike time sequences from 24 randomly selected trials of the same stimulus and the same cell, and the procedure was repeated 1000 times. The resulting samples were tested for their probability to be drawn from a uniform distribution.

We further verified that the two-sample KS test probabilities used to assess response consistency were sensitive to the different combinations of cells and stimuli. To this end, two random subsets of trials for pairs of neurons and stimuli were drawn and the Friedman test was applied to the median values of the resulting KS probabilities. In addition, one of the two subsets of trials was used to verify by means of the Wilcoxon test that the corresponding results were dependent on the probability distributions used in the comparisons.

### 2.6. Invariance analysis

The assessment of the stimulation method would not be complete if it was not possible to show that the transformation between the stimulation and the spike responses exhibited regularities, or invariant properties, independently from the attributes of each cell and each stimulus. With this aim in view, the average firing rates elicited by the 18 stimuli in the 18 cells were calculated and related to the velocity parameter that parametrized each stimulus family. As it was expected that the spike response should be associated, not only with the strain induced in the skin because the tissues and the mechanoreceptors have dynamic properties, but also with the magnitude of the strain rate which, by construction of the particular patterns we selected, was proportional to the speed of evolution of the stimuli, independently of their spatial organization. According to this hypothesis, the average firing rate elicited by a set of trials should depend on the speed of evolution, *v*, of stimuli for fixed spatial distributions according to power laws with exponents comprised between zero and one. A value of zero would indicate absence of dependence while a value of one would indicate proportionality.

### 2.7. Psychophysics

We controlled whether the different stimuli had perceptual value for humans. To this end, 10 participants (five female, five male) were asked to match the virtual stimuli described earlier with actual physical stimuli that were delivered manually by the experimenter. The latter comprised a representation for the ‘slip’ stimulus realized by sliding a glass surface with a Gaussian profile ridge (0.5 mm high, 3 mm wide) on the participants’ index fingers; a catch stimulus, represented by sliding a similar surface with a narrow trough (same profile, but inverted); a representation for the ‘roll’ stimulus realized by rolling a 30 mm plastic cylinder without slip against the finger and a representation of the ‘contact’ stimulus obtained by pressing a flat plastic surface against the participant's finger.

During 36 presentations in blocks of three (12 repetitions of each stimuli; 15 min experiment duration), the participants experienced the four physical stimuli presented in a randomized order and then felt three virtual potential equivalents (‘slip’ with *v* = 40 mm s^−1^, ‘roll’ with *v* = 30 mm s^−1^ and ‘contact’ with *v* = 50 mm s^−1^), also in a randomized order. The physical stimuli were identified by the order in which they were presented. After each virtual stimulus presentation, the participants reported which of the physical stimulus best matched what they felt. Throughout the experiment, the participants sat in a chair, wore a blindfold and the lights of the room were dimmed. All the participants found the task easy and did not report any fatigue.

## 3. Results

### 3.1. Spike timing

**Figure 5.**
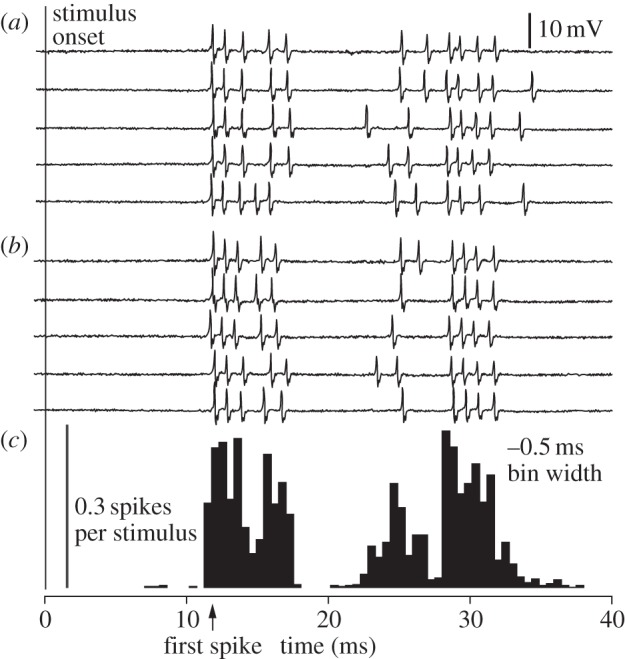
Cuneate cell response to ‘quadrant’ stimulus. (*a*) The five initial spike trains evoked by the stimulus. (*b*) Five spike trains randomly selected among the remaining 45. (*c*) Spike count histogram with 0.5 ms bin width obtained from 50 repetitions.

For a given stimulus, the responses were qualitatively similar, as exemplified by the five initial traces of figure 5*a* to be compared with five randomly selected traces of figure 5*b*. They show the spike response of a cell to the ‘quadrant’ stimulus, a pattern that is highly localized in space and in time. The first spike occurred 12.5 ± 1.00 ms (mean ± s.d.) after stimulus onset, over 50 repetitions. During these repetitions, the evoked responses consistently formed a pattern comprising four distinct groups of spikes. This pattern is evidenced by the 0.5 ms bin width histogram shown in figure 5*c*. In comparison, the response latency time to activation of the cuneate neurons using electrical stimulation of the primary afferents was 6.5–7.5 ms. Thus, the induced change in local skin strain was capable of generating sufficient depolarization in the skin mechanoreceptor to elicit a spike in its primary afferent within 5.5 ms.

**Figure 6.**
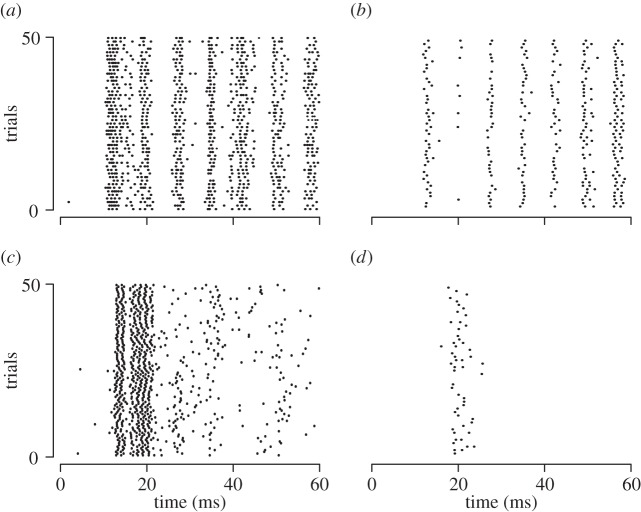
Samples of responses for one stimulus in raster form. (*a*–*c*) Examples of trials showing vigorous to moderate responses. (*d*) Weak response. (*a*) neuron 2-‘slip’ 160 mm s^−1^, (*b*) neuron 4-‘slip’ 160 mm s^−1^, (*c*) neuron 13-‘slip’ 160 mm s^−1^ and (*d*) neuron 6-‘slip’ 160 mm s^−1^.

The results of the two-sample KS similarity test are summarized in table 1 for *n* = 18 neurons. The median value of the probablities for two samples to be different was below 0.009 for all tests, meaning that for more than half of the neurons there were less than 1% of cases when the samples were different. The upper bound of the 95% CI (estimated using Wilcoxon statistics) was low as well, yet the highest values were significant in some cases. These cases correspond to the trials where the number of spikes was low and where the stochastic portion of the response dominates over the response triggered by the stimuli. Figure 6*a*–*c* illustrates typical trial responses. Figure 6*d* shows the case of a weak response to the same stimulus.

The fraction of trials that were significantly different from a response drawn from a uniform distribution and shown in table 2. The median value was always one, meaning that for at least half of the neurons the response patterns did not behave randomly throughout the entire battery of tests. If there are low values, it is because the number of spikes was also low. A weak stimulus for example ‘contact on’ at 10 mm s^−1^ elicited on average 1.2 spikes per trial.

**Table 2.** For each stimulus, fractions where the one-sample KS test reached an uncorrected threshold of *p* = 0.05, which was used as a criterion to decide that the pooled neural responses were different from random responses drawn from a uniform distribution.

stimuli	‘slip’					‘roll’			‘contact on’					‘contact off’				
velocity	10	20	40	80	160	5	10	20	10	20	50	100	200	10	20	50	100	200
KS median	1.00	1.00	1.00	1.00	1.00	1.00	1.00	1.00	1.00	1.00	1.00	1.00	1.00	1.00	1.00	1.00	1.00	1.00
KS lowest bound^a^	1.00	1.00	1.00	1.00	1.00	1.00	1.00	1.00	0.99	1.00	1.00	1.00	1.00	0.57	0.73	1.00	1.00	0.77
KS smallest value	1.00	1.00	1.00	1.00	1.00	0.33	0.93	1.00	0.73	0.23	0.54	1.00	1.00	0.06	0.23	0.50	0.49	0.62
mean spike count	114	83	61	38	21	305	168	92	56	33	20	14	9	61	35	22	14	7

^a^Of the 95% CI given by the Wilcoxon signed-rank test.

For the similarity tests (table 1), the median values of KS probabilities depended significantly on the stimulus type (Friedman test, *p*-value < 10^−5^) as well as on individual neurons (Friedman test, *p*-value < 10^−15^). The probability of rejecting the hypothesis that the KS probabilities were significantly smaller in the one-sample test compared with the two-sample test, as assessed by the Wilcoxon test, was very small (*p*-value < 10^−15^). Therefore, the distributions used in the comparisons were highly significant.

### 3.2. Specificity of the neural response to different stimuli

**Figure 7.**
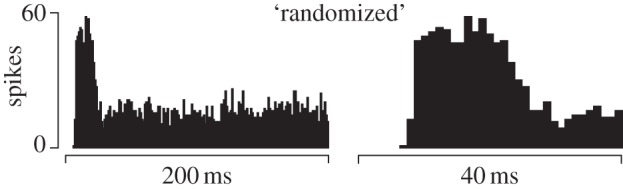
Cell response to the unspecific ‘randomized’ stimulus. (*a*) Complete histogram. (*b*) Initial portion enlarged showing the first 40 ms on the same timescale as in figure 5.

Inspection of individual responses showed that the stimulation technique was capable of producing qualitatively different responses in the same cell. The ‘randomized’ stimulus pattern produced a comparatively uniform response that lasted until the cessation of the stimulus (figure 7) with an initial burst of activity corresponding to the sudden activation of all the contact surfaces.

**Figure 8.**
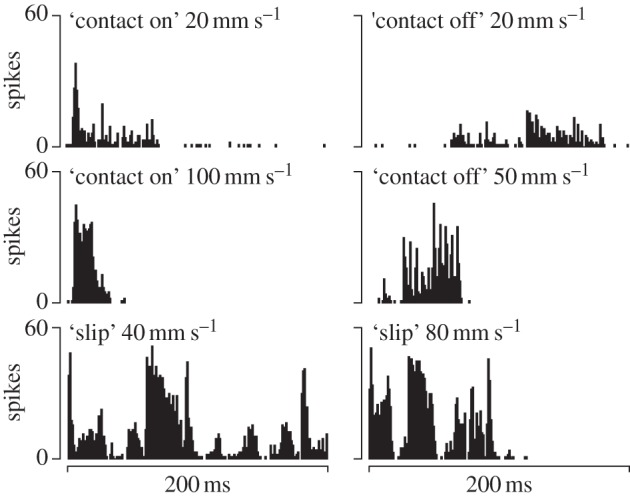
Histograms with 0.5 ms bin width of cell responses to several stimulus examples where the temporal evolution of a stimulus was changed.

With other stimulus patterns, as in the case of the ‘contact stimulus’ shown in figure 8, the same cell responded differently to the same spatial stimulus if it was produced at the same velocity but backward in time. The time-course of the response also changed when the velocity was changed in sign and in magnitude. For this cell, however, doubling the speed of the ‘slip’ stimulus did not markedly change the overall response, except of course, for its duration which was halved.

### 3.3. Response invariants

**Figure 9.**
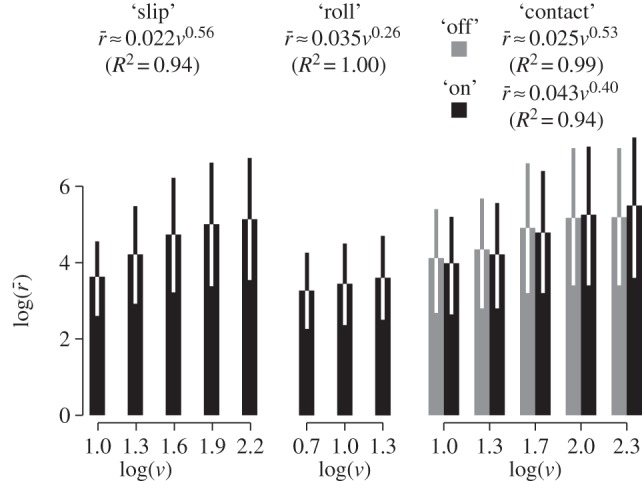
Averages and standard errors of firing rates, 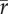, for all cells by stimulus family and speed, *v*. Each graph is associated with a fitted power law of the form *bv^a^*.

As seen in figure 9, across all cells, the average firing rate, 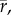 followed such power laws for all stimulus families. In particular, it could be verified from the ‘contact’ responses that the average firing rate depended strongly on the speed, *v*, of the stimulus but weakly on the direction of movement whereas, as shown earlier, the time-courses differed dramatically. Fitting power law relationships to the data gave exponents and magnitude factors that showed that the cells, as a whole, responded differently to the speed of evolution of the stimuli. The ‘roll’ stimulus family, in particular, exhibits a much weaker dependency on speed compared with the others.

**Figure 10.**
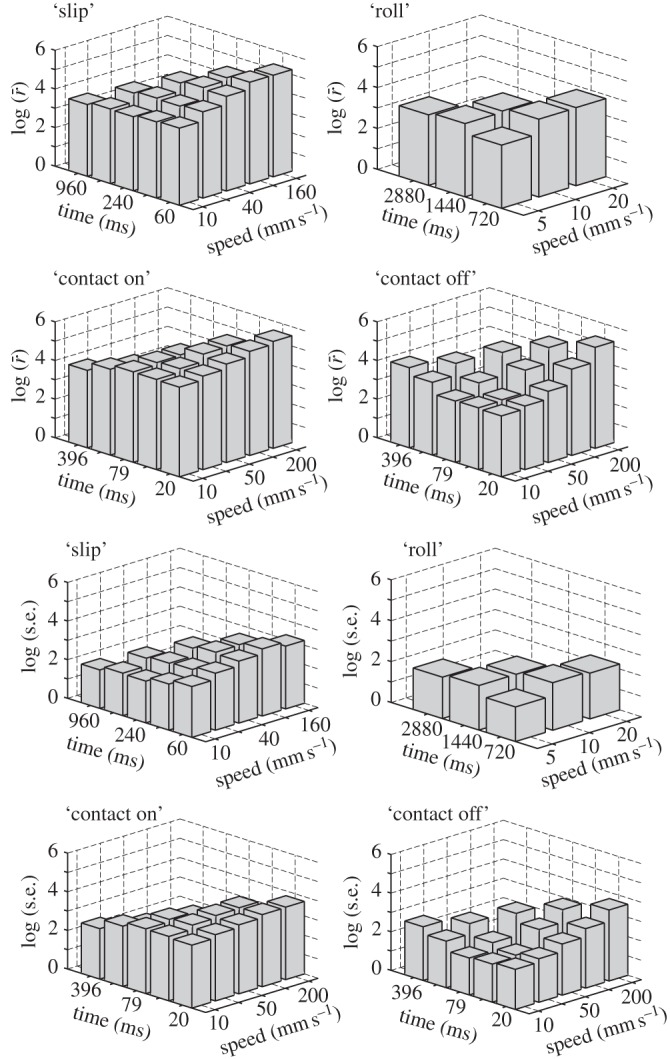
Averages and standard errors of firing rates for all cells by stimulus family separating the influence of the time window over which the rate was computed from the speed of temporal evolution of the stimuli.

To more finely represent the response to the spatio-temporal evolution of the stimulus families, the same data as in figure 9 are shown in figure 10 but plotted as a function of two quantities, the speed governing the temporal evolution of the stimuli and the duration of the time window over which the rate averages are obtained. If, as a whole, the cells responded only to the spatial characteristics of the stimuli, then the firing rates response would depend on time but not on speed. Alternatively, if the cells responded to the rate of evolution of the stimuli but not on their spatial properties, then the firing rates would depend on speed but not on time.

The cells where qualitatively less responsive to the spatial characteristics of the ‘slip’ stimulus, in this case the position in space of the virtual edge, and instead exhibited sensitivity to speed. There was a contrasting trend for the ‘contact on’ stimulus. The cells, as a whole, were weakly sensitive to speed but showed great sensitivity to the spatial characteristic of the stimulus which had a different spatial profile in the early stages compared with the later stages. Interestingly, the ‘contact off’ stimuli elicited responses that were highly sensitive to both space and speed. Lastly, the ‘roll’ stimulus elicited a weakly tuned response showing a preference for a certain combination of duration and speed.

The neuronal responses were quantified by their average firing rate within stimulus conditions. In 11 out the 18 conditions, the responses deviated significantly from normal distributions (*p*-value < 0.05, Shapiro–Wilk test). The effect of speed on the neurons' average firing rate could still be assessed using the Friedman test applied to the average firing rates for different neurons and different stimulus velocities within the same stimulus families, ‘contact on’, ‘contact off’, ‘slip’ and ‘roll’. High statistical significance of the effect of velocity was found in all cases (*p*-value < 0.001).

### 3.4. Psychophysical results

As indicated by the results in table 3, the ‘contact’ stimulus was dominantly associated by the participants to an actual contact, and so was the ‘roll’ stimulus. The ‘slip’ stimulus was also strongly associated with either the slipping ridge or the slipping trough with a net preference for the ridge.

**Table 3.** Confusions matrix between physical and virtual stimuli, *N* = 10 subjects. The mean and standard deviation of the percentage of cases when every virtual stimulus was associated with every physical stimulus.

	physical			
virtual	slip-trough	slip-ridge	roll	contact
‘slip’	28.0 ± 14.8	60.8 ± 25.2	6.7 ± 14.0	4.2 ± 9.0
‘roll’	16.7 ± 18.4	1.7 ± 5.3	81.6 ± 20.0	0 ± 0
‘contact’	2.5 ± 5.6	4.2 ± 8.0	0.8 ± 2.6	92.5 ± 16.0

## 4. Discussion and conclusion

Haptics involves mechanical interaction with objects, but as the skin is a mechanically deformable medium with slow time constants [12,13], it has been all but impossible to construct reliable models of the activation of the skin sensors, let alone of the central representation of cutaneous inputs. Our findings show that the stimulation method that we have described is able to induce consistent levels of skin strain varying through space and through time. Stimulation by differential traction greatly reduced the variations that result from the visco-elastic properties of the skin, owing to a large and stable support surface that provides a reliable boundary condition able to specify the displacement of the tissues everywhere in the vicinity of the stimulated mechanoreceptors.

Differential traction stimulation, designed to overcome the complications owing to the mechanics of the interface between the skin and the stimulator interface, was shown to generate highly reproducible spike responses in cuneate neurons, which represent the brain's first stage of integration of tactile information. Using a family of spatio-temporal skin stimuli, we further demonstrated that different spatio-temporal stimuli elicited different time-courses of spike responses in the same neuron.

As a whole, the responses exhibited strong regularities that support the idea that cuneate neurons, which each receive a large number of monosynaptic inputs from primary skin afferents [35,36], respond to a combination of strain and strain rate induced in the tissues present in their respective receptive fields, while remaining sensitive to the spatial organization of the individual stimuli. These regularities were reflected in the high level of human psychophysical performance in identifying the different families of stimuli.

The application of differential traction to the characterization of cuneate neurons responses provided us with a direct readout of the activation of the primary afferents present in their receptive fields. Its application need not be limited to such experiments nor, indeed, to micro-neurography in the peripheral or the central nervous system. The presently employed stimulation device, having very little metal in its construction and consuming little power, is naturally compatible with most neural activity imaging methods, prominently including functional magnetic resonance imaging and electro-encephalography. For magneto-encephalography, passive or active shielding precautions would have to be taken due to the necessary presence of currents in relative proximity of the magnetometers.

We conclude that the presently demonstrated skin stimulation technique, with its versatility in delivering a wide range of precisely controlled spatio-temporal stimuli, could be a valuable tool for the study of the differentiated processing of different classes of tactile inputs in the somatosensory systems of the brain.

## Figures and Tables

**Figure 11.**
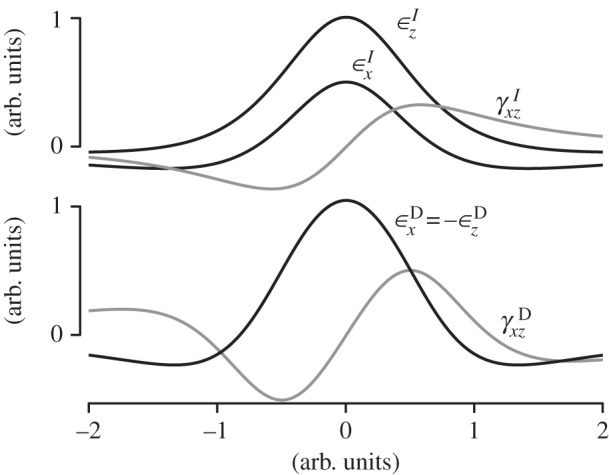
Comparison of strain distributions, indentation, *i*, versus differential traction, *d*, for *z*_0_ = 1.00 and *d* = 0.5. Extension–compression components (black lines) and shear components (grey lines).

**Figure 12.**
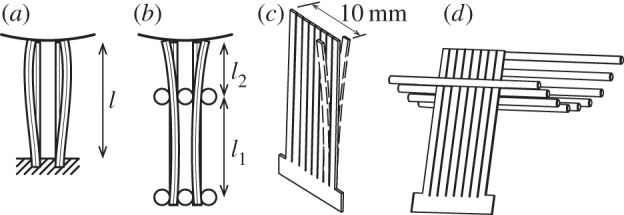
Device construction. (*a*) A pair of bending piezoelectric cantilevers used to create two adjacent traction surfaces on the skin are always either too weak or do not deflect sufficiently. (*b*) By contrast, dual pinned cantilevers can trade-off stiffness and deflection. They can be designed to cause up to 30% of strain in glabrous skin and remain of small size overall [30]. (*c*) Packs of eight benders cut from single piezoelectric slabs. (*d*) The assembly of eight slabs results in a array of 8 × 8 traction surfaces, here conforming to the curvature of digital pads to minimize initial contact deformation.

**Figure 13.**
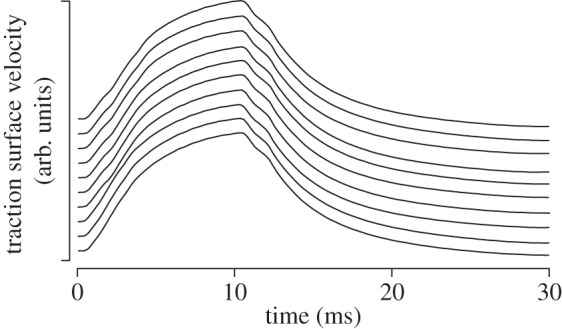
Ten experimental pulse responses of one traction surface. Velocity was measured by a high-fidelity phono pickup, a sensitive velocity sensor.

## Notes

The experimental procedure was approved by the ‘Comité de protection des personnes Ile-de-France II’. The experimental procedures were approved in advance by the Malmö/Lund Animal Research Ethics Committee (permit number and approval-ID: M05-12).

## Appendix A. Similarity between indentation and differential traction

The stresses induced by a line load pushing on the boundary of an infinite half-space with a lineic force, *P*, extending along the *y* direction, where *z* is in the depth direction, and *x* is in the tangential direction, are for a fixed depth, *z*_0_ [37].

Assuming Hooke's law in the *x*–*z* plane,  , and for a Poisson ratio, *v*, of 0.5 corresponding to an incompressible material (water saturated tissues), we obtain the subsurface strains owing to indentation,

Now, for the differential action of a pair line loads separated by distance 2*d* acting in the lateral direction with lineic force, *Q*, the following expressions were found [13],

Normalizing these expressions to unit multiplicative factors, to a depth of *z*_0_ = 1 arb. unit, and taking the distance between the differential line tractions to be 0.5 arb. units shows, graphically and without detailed analysis, the similarity of the strain patterns (figure 11). This observation shows the manner in which differential traction, at a proper distance beneath the skin surface, can replicate the effect of the interaction with surfaces. A more detailed analysis is in [13], where it is shown in particular that the expression *AE* ∝ *Q*, where *E* is the Young's modulus of the material and *A* the intensity of an equivalent indentation, holds. This formula expresses that, given a certain material and a certain contact surface spacing, differential traction can replicate, within limits, any indentation intensity. This property reduces the geometrical design problem of the stimulator to selecting proper spacing between contact surfaces which affects performance independently from the material properties of the skin. The skin properties impact only the required mechanical impedance and saturation level of the actuation.

## Appendix B. Device construction and performance

Tactile stimulators act through the surface of the skin, thus their action must be expressed in the form of variations of surface strain energy density. In the spatial domain, tactile display performance is expressed in terms of lineic forces because the gradient of surface strain energy density (in J m^−2^) with respect to surface strain is expressed in N m^−1^. The display could achieve 0.2 N mm^−1^, see discussion in [30].

Lateral traction on the surface of the skin was achieved through the action of piezoelectric bimorph benders. Physical scaling laws preclude that ordinary cantilevers mechanics parametrized by a length, *l*, (figure 12*a*, free deflection ∝ *l*^2^, stiffness ∝ *l*^3^) can achieve sufficient strain in skin for any geometry of piezoelectric benders [13]. Dual hinged cantilevers, figure 12*b*, however, can trade-off two design parameters, *l*_1_ and *l*_2_, and are much stiffer than ordinary cantilevers of the same material and for the same free deflection [30]. An actuated contact surface was realized by manufacturing sets of cantilevers from a single slab of piezoelectric bimorph benders, figure 12*c*, then stacking and spacing them, figure 12*d*, in a structural support and encasing it in a protective cover, as shown in figure 4*a*,*b*.

Because of its small size, mechanical simplicity and utilization of flexures for movement guidance, the device that we employed (Tactile Labs Inc.) was capable of high temporal resolution; the frequency of its lowest structural mode was higher than 1.0 kHz [30]. The contact surfaces were spaced each 1.2 mm, corresponding to regions of individually stimulated skin patches smaller than 1 mm^2^. The display head, figure 4*a*, comprised structural elements realized from printed circuit elements which also served for electrical signal routing and which supported an on-board, high-speed 64-channel analogue demultiplexer–amplifier subsystem. Figure 13 shows for one actuated traction surface 10 responses to a 10 ms pulse command. The aforementionned 0.58 ms system delay can be seen in combination with a characteristic first-order response dominated by the analogue 100 Hz reconstruction filter. Small residual structural oscillations can be seen in the response which are damped out after about 2 ms after the step input transients. None of the stimuli that we used included step transients.

## Appendix C. Preparation

Adult cats were prepared as previously described [38,39]. After initial anaesthesia with propofol (Diprivan, Zeneca), the animals were decerebrated at the intercollicular level, and the anaesthesia was discontinued. The animals were artificially ventilated, and end-expiratory CO_2_, blood pressure and rectal temperature were monitored and maintained within physiological limits. Mounting in a stereotaxic frame, drainage of cerebrospinal fluid, creation of a pneumothorax and clamping of the spinal processes of a few cervical and lumbar vertebral bodies served to increase the mechanical stability of the preparation.

## Appendix D. Recordings

The general procedures for this preparation have been described previously [39,40]. To obtain cuneate neuron recordings, we exposed the dorsal part of the lower brainstem by removing muscles overlying the junction between the skull and the first cervical vertebra. Parts of the caudal skull bone and the rostral C1 vertebra were also removed to improve access to the neural tissue. To improve stability, the exposed neural tissue was covered with agarose (2% dissolved in saline). Viewed through a stereo-microscope, the anatomical location of the cuneate nucleus is readily visible from the surface, with the landmarks of the obex and the groove between the cuneate nucleus and the spinal trigeminal nucleus being particularly helpful. The electrodes were inserted into the dorsal part of the nucleus, and neurons with receptive fields on the glabrous pads were searched for. Once a recording from such a neuron was obtained, the paw was mounted on the stimulation device with the pad that contained the receptive field centred on the active surface of the stimulator.
